# Are AMPA Receptor Positive Allosteric Modulators Potential Pharmacotherapeutics for Addiction?

**DOI:** 10.3390/ph7010029

**Published:** 2013-12-30

**Authors:** Lucas R. Watterson, M. Foster Olive

**Affiliations:** 1 Department of Psychology, Behavioral Neuroscience Area, Arizona State University, Tempe, AZ 85287, USA; 2 Interdisciplinary Graduate Program in Neuroscience, Arizona State University, Tempe, AZ 85287, USA

**Keywords:** AMPA PAMs, positive allosteric modulators, extinction, addiction

## Abstract

Positive allosteric modulators (PAMs) of α-amino-3-hydroxy-5-methyl-4-isoxazolepropionic acid (AMPA) receptors are a diverse class of compounds that increase fast excitatory transmission in the brain. AMPA PAMs have been shown to facilitate long-term potentiation, strengthen communication between various cortical and subcortical regions, and some of these compounds increase the production and release of brain-derived neurotrophic factor (BDNF) in an activity-dependent manner. Through these mechanisms, AMPA PAMs have shown promise as broad spectrum pharmacotherapeutics in preclinical and clinical studies for various neurodegenerative and psychiatric disorders. In recent years, a small collection of preclinical animal studies has also shown that AMPA PAMs may have potential as pharmacotherapeutic adjuncts to extinction-based or cue-exposure therapies for the treatment of drug addiction. The present paper will review this preclinical literature, discuss novel data collected in our laboratory, and recommend future research directions for the possible development of AMPA PAMs as anti-addiction medications.

## 1. Overview of Drug Addiction: The Need for Better Pharmacotherapeutics

Drug addiction is a chronically relapsing disorder principally characterized by the uncontrollable drive to obtain and failure to limit the use of drugs despite adverse, often severe, consequences. In addition, there is a concomitant loss of interest in engagement in other activities such as work, school, dependent care, and social gatherings, to name a few [1,2]. Addiction casts a wide net of consequences, adversely affecting not just the health and well-being of individual users, but devastating families, straining healthcare resources, significantly damaging environments, and imparting a significant economic and medical burden to society as a whole [3]. Globally, the use of addictive drugs is responsible for nearly 10% of the total disease burden [4]. In the United States, substance abuse is the leading preventable cause of death, illness, and disability [3]. When factoring in the influence of treating drug-related health problems, crime, loss of productivity due to disability and withdrawal from the workforce, and premature death, the economic burden of drug addiction to the US is estimated to exceed half a trillion dollars annually [5]. In 2011, 21.6 million persons in the U.S. aged 12 or older needed treatment for alcohol and illicit drug use disorders [6]. However, only 2.3 million received treatment, with the other 19.3 million declining help largely because of the costs and inconvenience of treatment [6]. Currently, the prevailing treatment approaches consist of traditional cognitive-behavioral therapies, self-help and social support programs, adjunctive treatment with approved medications (when applicable), and/or some combination thereof. However, only a small number of approved addiction pharmacotherapeutics exist, and only for nicotine, opioid, and alcohol addiction [7]. For addiction to psychostimulants such as cocaine and methamphetamine, there are currently no approved medications [8]. To make matters worse, even with the best treatment interventions, most individuals (up to 90% for some drugs) will relapse within 12 months of discontinuing drug use [9,10]. Given these severe consequences to users, families and society, the large number of individuals declining much needed treatment, and high rates of relapse even with treatment, there is a significant need for more effective treatment strategies designed to decrease the probability of relapse. One promising, yet largely untested, approach is the use of pharmacobehavioral strategies designed to reverse drug-induced impairments in executive functioning [7]. Here we hypothesize one such approach involving the combination of a class of nootropics (cognitive enhancers) known as AMPA PAMs with behavioral strategies known as cue-exposure therapy or extinction training.

## 2. “Addiction” *versus* “Substance Dependence”

Before discussing neural and behavioral aspects of addiction in more detail, it is important to clarify what we specifically mean by the term “addiction”. Over the last four decades, the term “substance dependence” has been used diagnostically by the American Psychiatric Association and World Health Organization, as it was believed to be a more neutral term than addiction (which was considered pejorative) [11,12,13,14]. Despite this, in the professional literature these terms have largely been used synonymously. However, as argued recently, they are not necessarily equivalent [12,13,14]. Many experts believe the use of “substance dependence” was a mistake and prefer “addiction” for a number of reasons [15,16]. First, experts agree that “addiction”, first and foremost, refers to the aberrant behaviors (compulsive drug-seeking and/or -taking) of disordered drug use. Second, “substance dependence”, but not “addiction”, is often construed as describing physical symptoms (“physical dependence”) such as tolerance and withdrawal that develop with the use of most drugs that affect the central nervous system. Thus, while the presence of these physical symptoms usually manifest with compulsive drug use, they are distinct phenomenon from the uncontrolled drug-seeking behavior that is the primary, necessary, and sufficient defining characteristic of the disorder. In addition, withdrawal commonly follows the cessation of use of therapeutic drugs that do not support compulsive use (e.g., antidepressants). While these semantics may seem trivial, it is has been argued that the use of “dependence” has led to unforeseen consequences such as under-treatment of pain disorders (where doctors express concern about patients becoming “addicted” to prescription opioids) and, at least superficially, appears to have narrowed the medications development focus toward drugs designed to alleviate withdrawal symptoms, reduce drug craving, and/or block rewarding effects of drugs. Attempts to design medications to rescue impaired executive functioning have only recently become a central focus in addiction research [17]. However, despite decades of “substance dependence” as the preferred diagnostic term, the term “substance use disorder”, under the category of “Addiction and Related Disorders”, has supplanted “substance dependence” in the fifth (and latest) version of the Diagnostic and Statistical Manual of Mental Disorders (DSM-V) [14]. Thus, in this review we will utilize the term “addiction” [18].

### 2.1. “Bottom-up”: Subcortical Neuroplasticity Drives the Development of Habitual Drug-Seeking Behavior

Addiction begins with controlled, episodic use motivated primarily by the positive reinforcing and rewarding effects of the drug [19]. These hedonic effects, like those of natural reinforcers (*i.e.*, food, water, sex, *etc.*), are predominantly mediated by increased dopamine (DA) transmission from neurons in the ventral tegmental area (VTA) of the midbrain to the ventral striatum (nucleus accumbens, NAc) [19]. This VTA → NAc pathway is generally considered to be the final common “reward” pathway for all reinforcers, both drug and otherwise [20]. DA transmission is also increased in other regions such as the amygdaloid complex (Amyg), ventral pallidum, hippocampus and prefrontal cortex (PFC) [20], which are believed to play more distinct roles in executive function, the formation of associations between drugs and external and interoceptive cues, and modulation of goal-directed behaviors [21]. With repeated exposure to rewarding or reinforcing stimuli, DA transmission in these circuits leads to cellular alterations that regulate how the organism behaves in the presence of motivationally relevant environmental stimuli, and mediate the establishment of adaptive responses necessary for acquiring future rewards or reinforcers [22,23]. When a natural reward or reinforcer is consumed, DA transmission in transiently activated and progressively diminishes with repeated exposure. Over time, neutral stimuli become conditioned reinforcers which themselves increase DA transmission, predicting the event and motivating the organism to engage in appropriate behavioral responses [19,24]. Thus, mesolimbic DA transmission both (1) initially signals the occurrence of motivationally relevant events and (2) later predicts the event from associated cues in order to engage in efficient goal-directed behaviors. With abused drugs, unlike natural reinforcers, increased DA transmission is robust, long-lasting, and *pathologically* reinforcing [20]. With repeated drug use, associations between the drug and previously neutral environmental stimuli (cues) become *exceedingly* salient conditioned reinforcers (associative “overlearning”) which can lead to craving and drive subsequent drug-seeking. Furthermore, with repeatedly reinforced drug-seeking events, this behavior becomes automatic, prepotent, and compulsive (instrumental “overlearning”) [25]. Thus, drugs of abuse “hijack” the subcortical systems that subserve normal motivational learning, and the combination of these “overlearning” processes produce lasting neuroadaptations in DA transmission that progressively lead to an escalated cycle of maladaptive (habitual) drug use [26].

Historically, researchers have thought that these neuroplastic changes mediated the transition from episodic to compulsive drug use and addiction [27,28]. However, research has shown that these subcortical neuroplastic changes alone are not fully capable in mediating the progression to compulsive drug use. Numerous lines of evidence in the last two decades, from both human neuroimaging and preclinical animal studies, have revealed that repeated drug use also disrupts prefrontal cortical functioning, resulting in a loss of executive functioning and “top-down” inhibitory control that, under normal circumstances, overrides habitual responding when exposed to adverse consequences [2,29,30,31]. Thus, drug addiction develops from a combination of subcortical alterations that drive automatic, habitual responding with a lack of top-down inhibitory control that regulates behavior in response to negative consequences. Given that most attempts to develop pharmaco-therapeutics for addiction have predominantly targeted only the subcortical reward systems (attempting to reduce craving or block rewarding and reinforcing effects of the drug), it is not surprisingly that the vast majority of compounds tested have failed to adequately reduce relapse rates, and only a few approved anti-relapse medications exist (and only for nicotine, alcohol, and opioids).

### 2.2. “Top-down”: Repeated Drug-Induced Insults to Prefrontal Cortices Impairs Executive Functioning

The PFC is responsible for many higher-order cognitive processes, often collectively referred to as executive functions, such as decision-making, response inhibition, planning, working memory, and attention [7,29,32]. As mentioned previously, in addition to increased DA signaling in the mesolimbic reward pathway, drugs of abuse also increase DA transmission in the PFC [33]. Evidence suggests that, while acute drug effects can increase PFC activity and improve cognitive functioning, repeated drug exposure leads to compensatory changes that subsequently both biases attention toward drug-related stimuli and impairs multiple domains of executive functioning (for a comprehensive review of drug-induced impairments in executive function domains, see [7,29,34]). In drug addiction, impaired functioning in these domains, combined with attention biased towards drug-related stimuli, culminates in the inability of the PFC to effectively exert “top-down” inhibitory control over habitual drug-seeking behavior [27,35]. While “bottom-up” DA transmission is responsible for innervating prefrontal regions, reciprocal “top-down” signaling from the PFC is mediated by the excitatory neurotransmitter glutamate [25,36,37]. In recent years, addiction research has begun to reveal that changes in glutamatergic signaling within corticostriatal and corticolimbic circuits where DA terminals are embedded are essential in mediating drug reward, reinforcement, and the transition to addiction [36,38,39,40], revealing new potential targets for addiction pharmacotherapeutics [17,41,42].

## 3. Glutamatergic Mechanisms in Memory Formation: A Brief Overview

Glutamate is the main excitatory neurotransmitter in the mammalian brain and responsible for approximately 70% of the chemical transmission in the central nervous system [25]. Glutamate binds to two major classes of receptors; ionotropic glutamate receptors (*N*-methyl-d-aspartate (NMDA), α-amino-3-hydroxy-5-methyl-4-isoxazolepropionic acid (AMPA), and kainate receptors) which mediate fast excitatory transmission, and metabotropic glutamate receptors (mGluR1-8) which mediate slow modulatory transmission through G-protein mediated signaling pathways [43]. At the cellular level, learning produces changes in excitatory glutamatergic transmission such as long-lasting increases in synaptic strength and postsynaptic current amplitudes, increasing the efficacy of communication between nerve cells. These changes, known as long-term potentiation (LTP), are generally accepted to be the cellular basis for memory formation and storage [44]. While there is an overwhelming amount of evidence suggesting that each of the glutamate receptor types play a unique role in LTP and learning and memory [45], for the purposes of this review, focus will be placed on ionotropic AMPA receptors (for a more comprehensive review on glutamate mechanisms and LTP, see [43,46]). Both early and late phases of LTP require AMPA receptors. First, signaling through AMPA receptors is necessary to slightly depolarize the membrane to approximately −50 mV, at which point the Mg^2+^ block is released from NMDA receptors, allowing Ca^2+^ ions to enter the cell. While Ca^2+^ triggers multiple downstream effects including gene transcription and translation mechanisms that results changes in the levels of numerous synaptic proteins, it also causes an immediate non-genomic increase in AMPA receptor trafficking and insertion of AMPA receptors into the plasma membrane, increasing the size and strength of postsynaptic responses [47]. The long-lasting increase in postsynaptic AMPA receptors is thought to be necessary for the lasting LTP and memory formation. Thus, ligands that increase signaling through AMPA receptors facilitate LTP, learning and memory [48].

As mentioned above, while dopaminergic signaling appears necessary for initiating and reinforcing early drug use (positive reinforcement), glutamatergic mechanisms within mesocorticolimbic circuits have also emerged as primary mediators of the transition to compulsive drug use [25,37]. Specifically, lasting neuroadaptations in corticostriatal and corticolimbic glutamatergic transmission are thought to be largely responsible for the behavioral hallmarks of addiction including (1) the impaired ability to regulate the drive to obtain and use drugs, even in the face of adverse consequences, and (2) a propensity to relapse even after long periods of abstinence [27,28,30]. In the normal brain, when motivational relevant stimuli are encountered, corticolimbic glutamatergic circuits, comprised of the PFC, amygdala, NAc core and shell (NAcc and NAcs), interact and send relevant environmental information through the NAc to mesostriatal (sensorimotor) circuits involving the dorsal striatum, which in turn communicates with other basal ganglia regions such as the globus pallidus and substantia nigra. Together, these circuits process environmental stimuli in order to establish efficient, goal-directed behaviors. Following repeated drug reinforcement, the influence of corticolimbic glutamatergic projections from the PFC and amygdala into the NAc progressively diminishes, whereas sensorimotor glutamatergic transmission to the dorsal striatum becomes predominant, allowing responses to become automatic (*i.e.*, habitual) and allowing corticolimbic circuits to process other relevant stimuli [35,49]. However, if reinforcer contingencies change and responses fail to yield expected outcomes, engaged corticolimbic circuits function to both inhibit the prepotent response and signal the motor cortex to generate new adaptive responses [19,28]. In other words, following repeated drug reinforcement, the influence of corticolimbic glutamate projections from the PFC to the NAc on behavior progressively diminishes, whereas sensorimotor glutamatergic transmission to the dorsal striatum becomes predominant, resulting in more automatic and habitual behaviors. Thus, compulsive drug use develops from a combination of pathologically strengthened “habit” circuitry combined with impaired corticolimbic circuits, rendering drug addicts with impaired behavior regulation who are unable to inhibit drug-seeking behavior in the face of adverse consequences [28,30,50]. Given the pivotal role of excitatory transmission in these circuits, treatments aimed at rescuing or increasing behavioral regulation and/or impairing drug-related “habit” memories may be promising avenues for novel addiction treatments.

## 4. Extinction/Exposure Strategies: Rescuing Behavioral Regulation

One therapeutic approach that has shown some success (although moderate at best) in decreasing relapse by enhancing behavioral regulation is cue exposure therapy [51,52,53]. In this approach, clinicians attempt to extinguish (*i.e.*, “break”) the associations between drug craving, use, and drug-related stimuli (such as drug paraphernalia) by repeatedly exposing drug users to the drug-related stimuli in the absence of drug availability. In preclinical rodent models of addiction, this process is studied using the extinction-reinstatement paradigm [54]. Here, a rodent or nonhuman primate is allowed to intravenously self-administer (IVSA) a drug, with drug infusions simultaneously paired with discrete cues such as a light and/or tone. Following stabilization of drug-taking, animals undergo either extinction training (ET), where they are placed in the drug-taking context but the drugs is no longer available (and thus drug-cue associations cannot be further strengthened), or no ET where they remain in their home cage (forced abstinence) [54]. Subsequently, animals are tested for reinstatement (“relapse”) of drug-seeking by the presentation of drug-associated discrete cues, a small dose of the drug, or a stressful stimulus [54]. Historically, a common misconception about exposure and extinction therapies has been that the resulting decrease in responding occurs due to a process of “forgetting”. However, evidence suggests that extinction is instead a form of new learning that is highly context dependent [55]. This is evidenced by the fact that, despite a loss of responding during extinction procedures, responding will often re-appear spontaneously with time (spontaneous recovery), when the organism is placed back in the original drug-taking context(s) (renewal), or exposed to discrete cues not present during the extinction procedures (cue-induced reinstatement) [56,57,58]. These phenomena suggest that exposure and extinction strategies do not erase the original drug-seeking memory engram(s), but instead decrease drug-seeking by strengthening “top-down” inhibitory control circuits [41]. However, the inconsistent success rates of exposure and extinction therapies in humans is likely attributable to due to either: (1) a lack of proper use of extinction procedures due to misunderstandings about the underlying processes of extinction (*i.e.*, new learning *vs.* forgetting), (2) context-specificity issues (*i.e.*, lack of extinction training in the actual drug-taking context(s)), (3) lack of adequate exposure session time, (4) lack of utilization of highly salient drug cues, or other uncontrolled variables [52,59].

Despite mixed results at the clinical level, preclinical studies show that extinction training (ET) decreases reinstated drug-seeking when compared to forced abstinence procedures where animals simply remain in their home cage for a matched amount of time [60,61]. Furthermore, reinstatement following forced abstinence is primarily mediated by dorsal striatal “habit” circuitry [62], whereas ET engages prefrontal glutamate projections to the NAcs, implying that top-down behavioral regulation circuits are also recruited [40,63,64,65]. Furthermore, similar to human imaging studies that have shown that cue-induced drug craving is correlated with anterior cingulate activation [66], the homologous prelimbic cortex in rats [67], which sends glutamatergic projections to the NAcc, is responsible for initiating cue-induced drug-seeking [27,64,68,69,70]. In contrast, ET enhances glutamatergic transmission from the infralimbic cortex (ILC) to the NAcs, which is a critical locus for the storage and consolidation of extinction learning and subsequent inhibition of cue-induced drug-seeking [40,63,65,71]. Thus, these two parallel PFC-NAc projections are functionally dichotomous, and compete for control of signaling in the NAc to motor circuits that ultimately guide behavior [27,72]. In addition, ET leads to persistent changes in various plasticity-related proteins in the NAc. Specifically, ET upregulates the expression of the GluR1 and GluR2/3 subunits of the AMPA receptor in the NAcs, indicative of the emergence of an LTP-like “up” state in these specific pathways. Corroborating these effects, viral overexpression of these same subunits also decreases reinstatement of cocaine-seeking [60,73]. Conversely, viral overexpression of “pore-dead” GluR1 subunits in the NAcc potentiates reinstated cocaine-seeking [60,74]. Thus, either potentiation or increasing the number of AMPA receptors in the NAcs, antagonism or decreasing the number of AMPA receptors in the NAcc, or both, would theoretically tip the balance of glutamatergic signaling to the NAc back towards favoring of ILC-NAcs mediated inhibitory control.

## 5. Facilitating ILC-NAcs Glutamate Signaling

The fact that ET recruits “top-down” glutamatergic signaling that mediates and is responsible for the consolidation of extinction behavior has led to an increase in research focusing on these pathways as pharmacotherapeutic targets. Recent studies have shown that various glutamate receptor agonists or PAMs enhance the consolidation of both extinguished drug-seeking and increase markers associated with synaptic plasticity in the ILC-NAcs pathway [40,65,71], suggesting that pharmacological compounds that enhance activity or plasticity in this pathway have the potential to be novel therapeutic adjuncts to cue exposure therapies [41]. One promising class of glutamate ligands, called AMPA PAMs [75,76], are small molecules that, while displaying a wide range of structural differences, all enhance glutamatergic signaling through positive modulation of AMPA receptors [77]. The first AMPA PAMs were developed approximately two decades ago, and in the time since have been shown to improve learning, memory and/or cognition, in both humans and animal subjects, and in a variety of experimental designs, indicating their potential as broad spectrum pharmacotherapeutics [48,76,78,79,80,81]. AMPA PAMs work in an activity-dependent manner by maintaining the open-channel state of AMPA receptors after binding of an endogenous ligand (glutamate) [82]. AMPA PAMs decrease either the rate of desensitization or deactivation of the receptor, thereby increasing cation influx into the postsynaptic cell [83]. However, unlike orthosteric (competitive) glutamate receptor agonists which can produce severe unwanted side effects such as excitoxicity, AMPA PAMs only enhance endogenous activity and are less prone to adverse side effects [84] (but see below). For example, evidence shows that AMPA PAMs can facilitate learning and memory at doses that do not cause excitotoxic damage, a common occurrence with orthosteric agonists [85,86,87,88]. However, it has recently been reported that AMPA PAMs may be more excitotoxic at effective doses than previously thought [89]. Nonetheless, most published studies have reported that AMPA PAMs generally have a safe profile at effective doses [48,76].

## 6. AMPA PAMs and Addiction: Preclinical Studies

In recent years, a handful of animal studies have assessed the potential use of AMPA PAMs for the treatment of addiction. In the first study of this kind [71], rats were allowed to intravenously self-administer (IVSA) cocaine for two weeks in daily 2 h sessions using standard operant lever pressing procedures. Following self-administration, rats were first placed into brief (15 min sessions) extinction sessions for five days after which intracranial ILC injections of the AMPA positive modulator 2-[2,6-difluoro-4-({2-[(phenylsulfonyl)amino]ethyl}thio)phenoxy]acetamide (PEPA, 30 ng/side) or vehicle were administered immediately after the extinction session. PEPA is a GluR3/4 preferring AMPA PAM that primarily exerts its effects through attenuation of AMPA receptor desensitization [90]. Next, seven additional 2 h extinction sessions were conducted, after which no post-session PEPA infusions were given, in order to assess for retention of extinction learning. The results showed that ILC injections of PEPA facilitated extinction learning (*i.e.*, decreased presses on the lever that previously resulted in cocaine delivery) during the final two 15-min extinction sessions. Furthermore, PEPA-facilitated extinction also continued through the seven 2 h extinction sessions, as overall responding was significantly decreased for all remaining ET sessions.

In a follow-up study by the same research group [40], rats underwent cocaine self-administration procedures for 2 weeks. Following cocaine IVSA, rats were again placed into ET for at least 10 sessions and remained in extinction until responding decreased to a predetermined criteria (>25 lever presses in two consecutive sessions). Following extinction procedures, PEPA microinjections into the ILC (0.075 nmol/hemisphere) were administered immediately prior to testing for cue-induced reinstatement. Two reinstatement tests were given, with rats receiving either PEPA or vehicle in a randomized, counter-balanced design. The results demonstrated that PEPA significantly decreased cue-induced reinstatement of cocaine-seeking compared to vehicle. Importantly, this decrease was not due to alterations in general locomotor activity. In a subsequent experiment in this study, it was also shown that PEPA-mediated decreases in responding were reversed by microinjections of an AMPA receptor antagonist into the NAcs. Together, these studies demonstrate that ILC glutamate transmission to the NAcs mediates the expression and consolidation of extinction behavior in the reinstatement paradigm.

While these studies demonstrate that facilitating glutamatergic transmission in the ILC→NAcs pathway with AMPA positive modulators is promising, there are no published reports demonstrating that systemic administration of AMPA PAMs, which is more translationally relevant for the development of newer treatments for addiction, produces similar promising results. Some AMPA PAMs have been reported to possess the ability to induce the expression and secretion of brain-derived neurotrophic factor (BDNF) [48], a neurotrophin that among other things facilitates the induction and maintenance of LTP [91]. Thus, AMPA PAMs can be further characterized into BDNF-inducing (BDNF AMPA PAM) or non-BDNF-inducing (non-BDNF AMPA PAM) subtypes [77,92]. For example, previous work has revealed increased motor recovery following experimental stroke in rats following administration of the BDNF AMPA PAM CX1837 as compared to the non-BDNF AMPA PAM CX1739 [92], suggesting that BDNF-inducing AMPA PAMs may have superior therapeutic potential.

**Figure 1:**
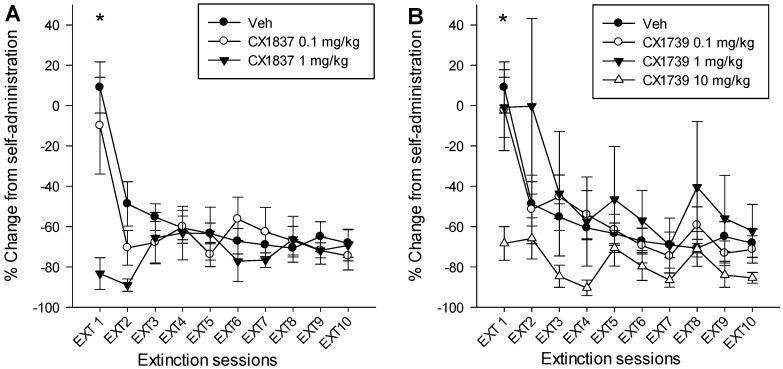
Male Sprague-Dawley rats were placed into 2 h daily methamphetamine IVSA-administration sessions for 10 days. Presses on an active lever produced methamphetamine infusions (0.05 mg/kg/infusion) on an FR1 schedule of reinforcement with a simultaneous 2 s light-tone stimulus complex. Following stable acquisition of methamphetamine IVSA, rats were placed into daily 2 h extinction sessions for 10 days during which active-lever presses no longer produced drug infusions or presentation of the stimulus complex. Twenty min prior to being placed into each extinction session, rats received intraperitoneal (i.p.) administration of either vehicle (Veh, 30% w/v 2-hydroxypropyl-β-cyclodextrin), CX1837 0.1 mg/kg (N = 6) CX1837 1 mg/kg (N = 12) CX1739 0.1 mg/kg (N = 7), 1 mg/kg (N = 7), or 10 mg/kg (N = 9). Vehicle treated rats (N = 20) were used for comparison for both CX1739 and CX1837. Data points represent the mean percent change (± SEM) from self-administration (mean of the final 2 days of self-administration procedures) for active lever presses. For CX1837, a mixed ANOVA analysis revealed a significant main effect of extinction session, F(9,306) = 5.78, *p* < 0.001, a significant extinction session x dose interaction, F(18, 306) = 2.77, *p* < 0.001, but no main effect of dose, F(2,34) = 1.32, *p* > 0.05. Post-hoc analyses revealed a significant reduction in responding on extinction day one by the 1 mg/kg dose of CX1837 *versus* vehicle, F(2,34) = 4.86, *p* < 0.05. No other measures were significantly different. For CX1739, a significant main effect of extinction session, F(9,351) = 15.180, *p* < 0.001, a significant extinction session X dose interaction, F(27,351) = 1.94, *p* < 0.004, but not a significant main effect of dose, F(3,39) = 2.60 *p* > 0.05. Post-hoc analyses revealed a significant reduction in responding on extinction day one by the 10 mg/kg dose of CX1739 *vs.* vehicle F(3,39) = 5.476, *p* < 0.003. No other measures were significantly different. All experimental procedures were conducted with the approval of the Institutional Animal Care and Use Committee at Arizona State University and according to the Guide for Care and Use of Laboratory Animals as adopted by the National Institutes of Health (NIH).

**Figure 2:**
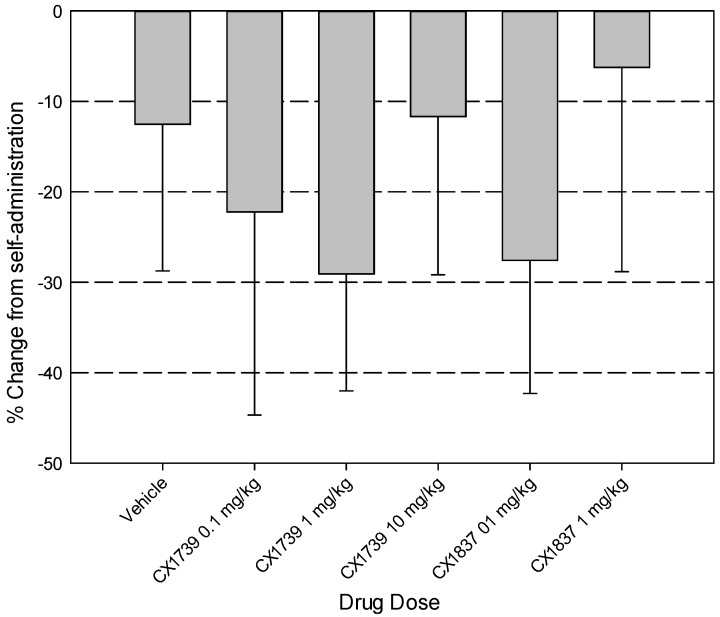
Following extinction sessions, rats were placed into cue-primed reinstatement procedures to assess the retention of extinction learning. Data points represent the mean percent change (±SEM) from self-administration (mean of the final 2 days of self-administration procedures) for active lever presses. A one-way ANOVA did not reveal significant differences between vehicle or any CX1837 doses (0.1 or 1 mg/kg, i.p.), F(3,39) = 0.161, *p* = 0.922, nor any significant differences between vehicle and of the doses of CX1739 tested (0.1, 1, or 10 mg/kg, i.p.), F(2,35) = 0.294, *p* = 0.747.

Recently, in collaboration with Cortex Pharmaceuticals (Glen Rock, NJ, USA), our laboratory has collected novel data on the effects of BDNF *vs.* non-BDNF AMPA PAMs on the extinction and reinstatement of methamphetamine-seeking behavior. Following two weeks of methamphetamine self-administration in rats, we systemically administered either the BDNF AMPA PAM CX1837 (0.1 and 1 mg/kg i.p., Figure 1a) or non-BDNFAMPA PAM CX1739 (0.1, 1, and 10 mg/kg i.p., Figure 1b) prior to ET sessions. Doses of these compounds were based upon recommendations from Cortex Pharmaceuticals and earlier reports of efficacious effects at similar doses [84,88] that do not alter generalized locomotor behavior [93]. Results revealed that systemic treatment with either CX1837 or CX1739 significantly facilitated extinction learning (reduction in active lever presses) on the first day of extinction tests (see Figure 1a,b). However, statistical analyses did not reveal any significant differences during any of the remaining extinction sessions or any main effects of drug type (CX1837 *vs.* CX1739). Furthermore, the reduction in responding seen during ET sessions unfortunately did not lead to significant reductions in cue-induced reinstatement of METH-seeking as seen previously following intra-ILC central injections of the AMPA PAM PEPA (Figure 2).

The observed lack of attenuated reinstatement by these AMPA PAMs is disappointing, especially in light of the aforementioned positive results observed with intra-ILC administration of PEPA following cocaine self-administration and ET. Reasons for the lack of apparent efficacy of either CX1739 or CX1839 in attenuating reinstatement may be attributable to the different drug reinforcers used (methamphetamine *vs.* cocaine), and it is possible that self-administration of these two psychostimulants produces differential effects on AMPA receptor and/or BDNF expression that may reduce the pharmacological effects of AMPA PAMs [94,95]. Alternatively, some studies have shown opposing prelimbic *vs.* ILC influences on the extinction and reinstatement of drug-seeking behavior [96], and it possible that potentiation of AMPA and/or BDNF signaling in both of these regions simultaneously following systemic AMPA PAM administration negates any effects of either of these compounds when acting in either region alone, as would be achieved by intracerebral administration. Thirdly, it is possible that CX1739 and/or CX1837 act on AMPA receptors containing subunit configurations that are different than those affected by PEPA (GluR3/4).

Nonetheless, these results do indicate that further research is needed to ascertain the potential therapeutic value of AMPA PAMs in the treatment of drug addiction. Specifically, future studies should examine factors such as drug reinforcer, BDNF *vs.* non-BDNF AMPA PAM utilized, and selectivity of these compounds for specific AMPA subunit composition and their neuroanatomical localization. Furthermore, it has recently been shown that a novel extinction paradigm, known as memory-retrieval extinction, leads to a reduction in cocaine, heroin, and alcohol reinstatement when compared to standard extinction training [97,98]. Furthermore, reductions in reinstatement are correlated with an upregulation of protein kinase M zeta (PKMζ), an atypical member of the protein kinase C family that is thought to be necessary and sufficient for long-term memories and LTP [99], although this has recently been challenged [100,101]. PKMζ, once synthesized, remains persistently active and maintains memories through an increase and maintenance of AMPA receptors in the post-synaptic membrane [99]. Thus, the attenuated reinstatement observed following memory-retrieval extinction procedures are likely mediated through upregulated AMPA receptor signaling, and further potentiation with AMPA PAMs may theoretically confer added benefits. This hypothesis, however, remains to be tested.

## 7. Discussion

Collectively, the results from the studies outlined above suggest that AMPA PAMs *may* have potential as pharmacological adjuncts to traditional cue-exposure therapies. However, the data thus far are rather limited, and this suggestion needs to remain hypothetical at this point until additional data are collected. Further studies with additional BDNF and non-BDNF AMPA PAMs, utilizing different drug reinforcers, and potentially additional extinction paradigms such as extinction-retrieval, are needed to provide firmer evidence of a therapeutic value of AMPA receptors in the treatment of addiction, such as novel pharmacological adjuncts to cue exposure therapy. Nonetheless, given that the ILC→NAcs glutamate pathway has been shown to mediate both the expression and consolidation of learned extinction of drug-seeking behavior, and AMPA PAMs exert their effects in an activity-dependent manner, a likely mechanism of the observed effects of AMPA PAM administration is potentiated glutamate transmission in this pathway. This hypothesis, while currently unconfirmed, suggests a facilitation of “top-down” inhibitory control over drug-seeking behavior. It is therefore possible that other AMPA PAM mechanisms may or may not contribute to the potential efficacy of these compounds in the context of drug addiction. While it has been suggested in previous work that positive effects AMPA PAMs may be mediated, in part, by alterations in neurotrophin (BDNF) signaling [48,76,78,92,102,103], our results did not reveal significant differences between the BDNF AMPA PAM CX1837 and the non-BDNF AMPA PAM CX1739, and thus does not suggest a significant role of BDNF signaling in the observed facilitated extinction effects. These results should be interpreted with caution however, and additional testing with other BDNF-inducing compounds is needed before definitive conclusions can be made.

## 8. Conclusions

Extinction-based cue-exposure therapies have shown limited success decreasing relapse in humans. However, evidence from preclinical studies suggests that extinction training, combined with AMPA PAMs treatment, under some circumstances, facilitates and consolidates extinction learning. Furthermore, under some circumstances AMPA PAM treatment also leads to attenuated cue-induced reinstatement of drug-seeking. These promising preclinical findings point to the need for future research aimed at assessing whether adjunct treatment with AMPA PAMs could potentially improve the success rate of cue-exposure therapies in humans.

## References

[1] Koob G.F., Sanna P.P., Bloom F.E. (1998). Neuroscience of addiction. Neuron.

[2] Kalivas P.W., Volkow N., Seamans J. (2005). Unmanageable motivation in addiction: A pathology in prefrontal-accumbens glutamate transmission. Neuron.

[3] Ericson N. Substance Abuse : The Nation’s Number One Health Problem.

[4] Harwood H., Bouchery E. (2004). The Economic Costs of Drug Abuse in the United States, 1992-2002.

[5] Fiscal Year 2008 Budget Request | National Institute on Drug Abuse http://www.drugabuse.gov/about-nida/legislative-activities/testimony-to-congress/2007/03/fiscal-year-2008-budget-request.

[6] Substance Abuse and Mental Health Services Administration, US Department of Health and Human Services Results from the 2011 National Survey on Drug Use and Health: Summary of National Findings.

[7] Sofuoglu M., DeVito E.E., Waters A.J., Carroll K.M. (2013). Cognitive enhancement as a treatment for drug addictions. Neuropharmacology.

[8] Sofuoglu M. (2010). Cognitive enhancement as a pharmacotherapy target for stimulant addiction. Addiction.

[9] Hendershot C.S., Witkiewitz K., George W.H., Marlatt G.A. (2011). Relapse prevention for addictive behaviors. Subst. Abuse Treat. Prev. Policy.

[10] Brandon T.H., Vidrine J.I., Litvin E.B. (2007). Relapse and relapse prevention. Annu. Rev. Clin. Psychol..

[11] American Psychiatric Association (2000). Diagnostic and Statistical Manual of Mental Disorders: DSM-IV-TR.

[12] Maddux J.F., Desmond D.P. (2000). Addiction or dependence?. Addiction.

[13] O’Brien C.P., Volkow N.D., Li T.-K. (2006). What’s in a word? Addiction *versus* dependence in DSM-V. Am. J. Psychiatry.

[14] O’Brien C. (2011). Addiction and dependence in DSM-V. Addiction.

[15] Ahmed S.H. (2010). Validation crisis in animal models of drug addiction: beyond non-disordered drug use toward drug addiction. Neurosci. Biobehav. Rev..

[16] Ahmed S.H. (2012). The science of making drug-addicted animals. Neuroscience.

[17] Kalivas P., Volkow N.D.N. (2011). New medications for drug addiction hiding in glutamatergic neuroplasticity. Mol. Psychiatry.

[18] American Psychiatric Association (2013). Diagnostic and Statistical Manual of Mental Disorders.

[19] Berridge K.C., Robinson T.E. (1998). What is the role of dopamine in reward: hedonic impact, reward learning, or incentive salience?. Brain Res. Brain Res. Rev..

[20] Feltenstein M.W., See R.E. (2008). The neurocircuitry of addiction: An overview. Br. J. Pharmacol..

[21] Hyman S.E., Malenka R.C. (2001). Addiction and the brain: the neurobiology of compulsion and its persistence. Nat. Rev. Neurosci..

[22] Spanagel R., Weiss F. (1999). The dopamine hypothesis of reward: past and current status. Trends Neurosci..

[23] Graybiel A.M. (2008). Habits, rituals, and the evaluative brain. Annu. Rev. Neurosci..

[24] Kalivas P.W. (2007). Neurobiology of cocaine addiction: implications for new pharmacotherapy. Am. J. Addict..

[25] Gass J.T., Olive M.F. (2008). Glutamatergic substrates of drug addiction and alcoholism. Biochem. Pharmacol..

[26] Cleva R., Gass J. (2010). Neuroanatomical structures underlying the extinction of drug-seeking behavior. Open Addict. J..

[27] Kalivas P.W., O’Brien C. (2008). Drug addiction as a pathology of staged neuroplasticity. Neuropsychopharmacology.

[28] Kalivas P., Davis K.L., Charney D., Coyle J.T., Nemeroff C. (2002). Neurocircuitry of addiction. Neuropsychopharmacology.

[29] Goldstein R.Z., Volkow N.D. (2011). Dysfunction of the prefrontal cortex in addiction: Neuroimaging findings and clinical implications. Nat. Rev. Neurosci..

[30] Kalivas P., Volkow N. (2005). The neural basis of addiction: A pathology of motivation and choice. Am. J. Psychiatry.

[31] Jentsch J.D., Taylor J.R. (1999). Impulsivity resulting from frontostriatal dysfunction in drug abuse: implications for the control of behavior by reward-related stimuli. Psychopharmacology.

[32] Miller E.K., Cohen J.D. (2001). An integrative theory of prefrontal cortex function. Annu. Rev. Neurosci..

[33] Koob G.F., Volkow N.D. (2010). Neurocircuitry of addiction. Neuropsychopharmacology.

[34] Field M., Cox W.M. (2008). Attentional bias in addictive behaviors: a review of its development, causes, and consequences. Drug Alcohol Depend..

[35] Kalivas P.W. (2008). Addiction as a pathology in prefrontal cortical regulation of corticostriatal habit circuitry. Neurotox. Res..

[36] Tzschentke T.M., Schmidt W.J. (2003). Glutamatergic mechanisms in addiction. Mol. Psychiatry.

[37] Kalivas P.W., Lalumiere R.T., Knackstedt L., Shen H. (2009). Glutamate transmission in addiction. Neuropharmacology.

[38] Gass J.T., Olive M.F.F. (2009). Positive allosteric modulation of mGluR5 receptors facilitates extinction of a cocaine contextual memory. Biol. Psychiatry.

[39] Peters J., Kalivas P.W., Quirk G.J. (2009). Extinction circuits for fear and addiction overlap in prefrontal cortex. Learn. Mem..

[40] LaLumiere R.T., Smith K.C., Kalivas P.W. (2012). Neural circuit competition in cocaine-seeking: Roles of the infralimbic cortex and nucleus accumbens shell. Eur. J. Neurosci..

[41] Cleva R.M., Gass J.T., Widholm J.J., Olive M.F. (2010). Glutamatergic targets for enhancing extinction learning in drug addiction. Curr. Neuropharmacol..

[42] Olive M.F., Cleva R.M., Kalivas P.W., Malcolm R.J. (2012). Glutamatergic medications for the treatment of drug and behavioral addictions. Pharmacol. Biochem. Behav..

[43] Niciu M.J., Kelmendi B., Sanacora G. (2012). Overview of glutamatergic neurotransmission in the nervous system. Pharmacol. Biochem. Behav..

[44] Lisman J., Yasuda R., Raghavachari S. (2012). Mechanisms of CaMKII action in long-term potentiation. Nat. Rev. Neurosci..

[45] Nicoll R.A., Roche K.W. (2013). Long-term potentiation: Peeling the onion. Neuropharmacology.

[46] Lamprecht R., LeDoux J. (2004). Structural plasticity and memory. Nat. Rev. Neurosci..

[47] Chang P.K.-Y., Verbich D., McKinney R.A. (2012). AMPA receptors as drug targets in neurological disease—Advantages, caveats, and future outlook. Eur. J. Neurosci..

[48] Lynch G., Gall C.M. (2006). Ampakines and the threefold path to cognitive enhancement. Trends Neurosci..

[49] Jog M.S. (1999). Building neural representations of habits. Science.

[50] Everitt B.J., Robbins T.W. (2005). Neural systems of reinforcement for drug addiction: from actions to habits to compulsion. Nat. Neurosci..

[51] Marlatt G.A. (1990). Cue exposure and relapse prevention in the treatment of addictive behaviors. Addict. Behav..

[52] Conklin C.A., Tiffany S.T. (2002). Applying extinction research and theory to cue-exposure addiction treatments. Addiction.

[53] Havermans R.C., Jansen A.T.M. (2003). Increasing the efficacy of cue exposure treatment in preventing relapse of addictive behavior. Addict. Behav..

[54] Epstein D.H., Preston K.L., Stewart J., Shaham Y. (2006). Toward a model of drug relapse: an assessment of the validity of the reinstatement procedure. Psychopharmacology.

[55] Bouton M.E. (2004). Context and behavioral processes in extinction. Learn. Mem..

[56] Bouton M. (2002). Context, ambiguity, and unlearning: Sources of relapse after behavioral extinction. Biol. Psychiatry.

[57] Crombag H.S., Bossert J.M., Koya E., Shaham Y. (2008). Context-induced relapse to drug seeking: A review. Philos. Trans. R. Soc. Lond. B Biol. Sci..

[58] Rescorla R. (2004). A Spontaneous recovery. Learn. Mem..

[59] Taylor J.R., Olausson P., Quinn J.J., Torregrossa M.M. (2009). Targeting extinction and reconsolidation mechanisms to combat the impact of drug cues on addiction. Neuropharmacology.

[60] Sutton M.A., Schmidt E.F., Choi K.-H., Schad C.A., Whisler K., Simmons D., Karaian D.A., Monteggia L.M., Neve R.L., Self D.W. (2003). Extinction-induced upregulation in AMPA receptors reduces cocaine- seeking behaviour. Nature.

[61] Fuchs R.A., Branham R.K., See R.E. (2006). Different neural substrates mediate cocaine seeking after abstinence *versus* extinction training: A critical role for the dorsolateral caudate-putamen. J. Neurosci..

[62] Di Ciano P., Robbins T.W., Everitt B.J. (2008). Differential effects of nucleus accumbens core, shell, or dorsal striatal inactivations on the persistence, reacquisition, or reinstatement of responding for a drug-paired conditioned reinforcer. Neuropsychopharmacology.

[63] Peters J., LaLumiere R.T., Kalivas P.W. (2008). Infralimbic prefrontal cortex is responsible for inhibiting cocaine seeking in extinguished rats. J. Neurosci..

[64] LaLumiere R.T., Kalivas P.W. (2008). Glutamate release in the nucleus accumbens core is necessary for heroin seeking. J. Neurosci..

[65] Knackstedt L.A., Moussawi K., LaLumiere R.T., Schwendt M., Klugmann M., Kalivas P.W. (2010). Extinction training after cocaine self-administration induces glutamatergic plasticity to inhibit cocaine seeking. J. Neurosci..

[66] Childress A., Mozley P. (1999). Limbic activation during cue-induced cocaine craving. Am. J. Psychiatry.

[67] Ongür D., Price J.L. (2000). The organization of networks within the orbital and medial prefrontal cortex of rats, monkeys and humans. Cereb. Cortex.

[68] McFarland K., Kalivas P.W. (2001). The circuitry mediating cocaine-induced reinstatement of drug-seeking behavior. J. Neurosci..

[69] Kalivas P.W., McFarland K. (2003). Brain circuitry and the reinstatement of cocaine-seeking behavior. Psychopharmacology.

[70] McFarland K., Lapish C.C., Kalivas P.W. (2003). Prefrontal glutamate release into the core of the nucleus accumbens mediates cocaine-induced reinstatement of drug-seeking behavior. J. Neurosci..

[71] LaLumiere R.T., Niehoff K.E., Kalivas P.W. (2010). The infralimbic cortex regulates the consolidation of extinction after cocaine self-administration. Learn. Mem..

[72] Kalivas P.W. (2009). The glutamate homeostasis hypothesis of addiction. Nat. Rev. Neurosci..

[73] Ghasemzadeh M.B., Vasudevan P., Mueller C., Seubert C., Mantsch J.R. (2009). Region specific alterations in glutamate receptor expression and subcellular distribution following extinction of cocaine self-administration. Brain Res..

[74] Bachtell R.K., Choi K.-H., Simmons D.L., Falcon E., Monteggia L.M., Neve R.L., Self D.W. (2008). Role of GluR1 expression in nucleus accumbens neurons in cocaine sensitization and cocaine-seeking behavior. Eur. J. Neurosci..

[75] Lynch G. (2002). Memory enhancement: the search for mechanism-based drugs. Nat. Neurosci..

[76] Lynch G. (2006). Glutamate-based therapeutic approaches: ampakines. Curr. Opin. Pharmacol..

[77] Arai A.C., Kessler M. (2007). Pharmacology of ampakine modulators: from AMPA receptors to synapses and behavior. Curr. Drug Targets.

[78] Lynch G., Palmer L.C., Gall C.M. (2011). The likelihood of cognitive enhancement. Pharmacol. Biochem. Behav..

[79] Swanson G. (2009). Targeting AMPA and kainate receptors in neurological disease: therapies on the horizon?. Neuropsychopharmacology.

[80] Black M.D. (2005). Therapeutic potential of positive AMPA modulators and their relationship to AMPA receptor subunits. A review of preclinical data. Psychopharmacology.

[81] Marenco S., Weinberger D.R. (2006). Therapeutic potential of positive AMPA receptor modulators in the treatment of neuropsychiatric disorders. CNS Drugs.

[82] Jin R., Clark S., Weeks A.M., Dudman J.T., Gouaux E., Partin K.M. (2005). Mechanism of positive allosteric modulators acting on AMPA receptors. J. Neurosci..

[83] ONeill M., Bleakman D. (2004). AMPA receptor potentiators for the treatment of CNS disorders. CNS Neurol. Disord..

[84] Christopoulos A. (2002). Allosteric binding sites on cell-surface receptors: novel targets for drug discovery. Nat. Rev. Drug Discov..

[85] Olney J.W. (1994). Excitatory transmitter neurotoxicity. Neurobiol. Aging.

[86] Staubli U., Rogers G., Lynch G. (1994). Facilitation of glutamate receptors enhances memory. Proc. Natl. Acad. Sci. USA.

[87] Mattson M. (2003). Excitotoxic and excitoprotective mechanisms. Neuromol. Med..

[88] Mehta A., Prabhakar M., Kumar P., Deshmukh R., Sharma P.L. (2013). Excitotoxicity: bridge to various triggers in neurodegenerative disorders. Eur. J. Pharmacol..

[89] Shaffer C.L., Hurst R.S., Scialis R.J., Osgood S.M., Bryce D.K., Hoffmann W.E., Lazzaro J.T., Hanks A.N., Lotarski S., Weber M.L. (2013). Positive allosteric modulation of AMPA receptors from efficacy to toxicity: The interspecies exposure-response continuum of the novel potentiator PF-4778574. J. Pharmacol. Exp. Ther..

[90] Sekiguchi M., Nishikawa K., Aoki S., Wada K. (2002). A desensitization-selective potentiator of AMPA-type glutamate receptors. Br. J. Pharmacol..

[91] Bramham C.R., Messaoudi E. (2005). BDNF function in adult synaptic plasticity: The synaptic consolidation hypothesis. Prog. Neurobiol..

[92] Clarkson A.N., Overman J.J., Zhong S., Mueller R., Lynch G., Carmichael S.T. (2011). AMPA receptor-induced local brain-derived neurotrophic factor signaling mediates motor recovery after stroke. J. Neurosci..

[93] Silverman J.L., Oliver C.F., Karras M.N., Gastrell P.T., Crawley J.N. (2013). AMPAKINE enhancement of social interaction in the BTBR mouse model of autism. Neuropharmacology.

[94] Bowers M.S., Chen B.T., Bonci A. (2010). AMPA receptor synaptic plasticity induced by psychostimulants: the past, present, and therapeutic future. Neuron.

[95] Ghitza U.E., Zhai H., Wu P., Airavaara M., Shaham Y., Lu L. (2010). Role of BDNF and GDNF in drug reward and relapse: A review. Neurosci. Biobehav. Rev..

[96] Willcocks A.L., McNally G.P. (2013). The role of medial prefrontal cortex in extinction and reinstatement of alcohol-seeking in rats. Eur. J. Neurosci..

[97] Xue Y.-X., Luo Y.-X., Wu P., Shi H.-S., Xue L.-F., Chen C., Zhu W.-L., Ding Z.-B., Bao Y.-P., Shi J. (2012). A memory retrieval-extinction procedure to prevent drug craving and relapse. Science.

[98] Zayra Millan E., Milligan-Saville J., McNally G.P. (2013). Memory retrieval, extinction, and reinstatement of alcohol seeking. Neurobiol. Learn. Mem..

[99] Sacktor T. (2010). How does PKMζ maintain long-term memory?. Nat. Rev. Neurosci..

[100] Volk L.J., Bachman J.L., Johnson R., Yu Y., Huganir R.L. (2013). PKM-ζ is not required for hippocampal synaptic plasticity, learning and memory. Nature.

[101] Lee A.M., Kanter B.R., Wang D., Lim J.P., Zou M.E., Qiu C., McMahon T., Dadgar J., Fischbach-Weiss S.C., Messing R.O. (2013). Prkcz null mice show normal learning and memory. Nature.

[102] Lauterborn J.C., Pineda E., Chen L.Y., Ramirez E.A., Lynch G., Gall C.M. (2009). Ampakines cause sustained increases in brain-derived neurotrophic factor signaling at excitatory synapses without changes in AMPA receptor subunit expression. Neuroscience.

[103] Lynch G. (1998). Memory and the brain: unexpected chemistries and a new pharmacology. Neurobiol. Learn. Mem..

